# Letter to the editor: a commentary on “Diagnostic accuracy of dual-energy CT Rho/Z maps for detecting secondary choledocholithiasis”

**DOI:** 10.1097/JS9.0000000000003147

**Published:** 2025-08-07

**Authors:** Yuqin Zhang

**Affiliations:** Department of Radiology, Suining Central Hospital, Suining, China

*Dear Editor*,

We read with great interest the recent study by Zhang et al^[1]^ titled “Diagnostic Accuracy of Dual-Energy CT Rho/Z Maps for Detecting Secondary Choledocholithiasis.” This study investigated the diagnostic performance of dual-energy CT (DECT) electron density (Rho) and effective atomic number (Z) maps in detecting secondary choledocholithiasis, suggesting their potential as alternatives to Magnetic Resonance Cholangiopancreatography (MRCP) for this purpose. This work represents a significant advancement in the noninvasive identification of isodense gallstones and demonstrates a breakthrough in DECT applications. We have commented the authors for their rigorous review.

The strengths of this study are as follows: First, it demonstrates that combining Rho and Z maps significantly enhances diagnostic performance compared to conventional CT, increasing the area under the curve (AUC) from 0.734 to 0.855 (*P*= 0.007). While maintaining a specificity of 95.7%, the sensitivity improves substantially (74.4% vs. 51.2%, *P*= 0.004). This addresses a long-standing clinical challenge, as conventional CT often fails to detect isodense gallstones, which accounted for 23.6% of the cases in this study. Decision curve analysis further confirmed that the Rho plus Z maps provided net benefits across a wide range of clinical risk thresholds (20%–95%), underscoring their utility in emergency decision-making. Second, the diagnostic performance of the Rho/Z map combination for choledocholithiasis is comparable to that of MRCP (AUC: 0.855 vs. 0.875, *P*= 0.526). This suggests that DECT Rho/Z maps could serve as a viable alternative for patients with contraindications to MRCP (e.g., metallic implants, claustrophobia) or in resource-limited settings.

We are also intrigued by the mechanistic explanation provided: Rho maps excel in identifying stones with higher material density (such as calcium bilirubinate), while Z maps complement this by visualizing cholesterol-rich stones through differences in effective atomic numbers. This synergistic effect highlights the value of multiparametric spectral imaging for distinguishing subtle tissue differences beyond traditional Hounsfield units.

While the study’s retrospective, single-center design and reliance on a specific vendor’s equipment are acknowledged limitations, the rigorous methodology, including adherence to the STARD criteria and TITAN guidelines, strengthens the validity of its conclusions. However, several areas require further investigation.

First, the study included a relatively high proportion of calcified stones (24.7%, 22/89) that were readily detectable on conventional CT. The core clinical challenge lies in the identification of isodense stones (23.6%, 21/89). We recommend supplementing the analysis with subgroup analyses stratified by stone density to clarify whether Rho/Z maps specifically enhance the detection rate of radiolucent stones, which is crucial for clinical application.

Second, while the manuscript states that Rho/Z maps improve diagnostic efficacy by detecting chemical composition differences (e.g., electron density and effective atomic number) between stones and bile, the current analysis primarily relies on qualitative assessment (e.g., visual comparison and consensus reading). This results in diagnostic criteria that are dependent on the radiologist’s subjective judgment. Future research could establish a Rho/Z database through large-sample, multi-center studies, supplement it with quantitative analysis to define standardized thresholds, and integrate machine learning to develop automated detection models, thereby enhancing the diagnostic objectivity.

Third, the suboptimal image quality of the Z-map (median Likert score: 3 vs. 5 for Rho/conventional CT; *P* < 0.001) was likely the primary reason for its inferior standalone performance. Future studies could consider technical strategies to improve the signal-to-noise ratio of the Z-map (e.g., noise-reduction algorithms or keV optimization).

Finally, this study utilized unenhanced CT to avoid contrast agent interference. However, its utility in the evaluation of an acute abdomen, where contrast-enhanced CT is often the gold standard, requires further validation. Exploring Rho/Z reconstruction based on contrast-enhanced DECT could significantly expand its clinical applicability.

In conclusion, Zhang et al were the first to apply DECT Rho/Z maps to the detection of choledocholithiasis, making a significant contribution to abdominal imaging. The combination of Rho and Z maps represents a promising advancement in the noninvasive diagnosis of secondary choledocholithiasis, with the potential to improve patient care and resource utilization. We believe that this work will inspire further research on spectral CT applications for biliary and other abdominal pathologies.

## Data Availability

All data analyzed or generated during this study are included in this article.
